# Use of Digital Technologies to Maintain Older Adults’ Social Ties During Visitation Restrictions in Long-Term Care Facilities: Scoping Review

**DOI:** 10.2196/38593

**Published:** 2023-02-10

**Authors:** Célia Lemaire, Christophe Humbert, Cédric Sueur, Céline Racin

**Affiliations:** 1 Magellan (EA 3713) iaelyon Université Jean Moulin Lyon 3 Lyon France; 2 Humans and Management in Society (UR 7308) École de Management Strasbourg Université de Strasbourg Strasbourg France; 3 Faculté des sciences de l’administration Université Laval Québec, QC Canada; 4 PSInstitut Strasbourg France; 5 Laboratoire interdisciplinaire en études culturelles (UMR 7069) Université de Strasbourg Strasbourg France; 6 Institut pluridisciplinaire Hubert Curien (UMR 7178), Centre national de la recherche scientifique Université de Strasbourg Strasbourg France; 7 Anthropolab, Ethics on experiments, Transhumanism, Human Interactions, Care & Society (EA 7446) Université Catholique de Lille Lille France; 8 Institut Universitaire de France Paris France; 9 Subjectivité, Lien Social et Modernité (EA 3071), Faculté de psychologie Université de Strasbourg Strasbourg France; 10 Centre de Recherche en Psychopathologie et Psychologie Clinique (EA 653), Institut de psychologie Université Lumière Lyon 2 Lyon France

**Keywords:** social isolation, COVID-19 pandemic, remote care, nursing homes, social ties, digital devices, older adults

## Abstract

**Background:**

Digital technologies were implemented to address the disruption of long-term care facility residents’ socialization needs during the COVID-19 pandemic. A literature review regarding this topic is needed to inform public policy, facility managers, family caregivers, and nurses and allied health professionals involved in mediating the use of digital devices for residents’ social ties.

**Objective:**

Our study outlines key concepts, methodologies, results, issues, and gaps in articles published during pandemic-related visitation restrictions.

**Methods:**

Following the PRISMA-ScR (Preferred Reporting Items for Systematic Reviews and Meta-Analyses Extension for Scoping Reviews) protocol, a scoping review was conducted by searching 3 database aggregator platforms (EBSCO, ProQuest, and PubMed) for studies published in peer-reviewed journals from early 2020 to the end of June 2021, when the most stringent restrictions were in place. We included qualitative and quantitative studies, reviews, commentaries, viewpoints, and letters to the editors in French or English focusing on digital technologies aiming to support the social contact of residents in long-term care facilities during pandemic-related visitation restrictions.

**Results:**

Among 763 screened articles, 29 met our selection criteria. For each study, we characterized the (1) authors, title, and date of the publication; (2) country of the first author; (3) research fields; (4) article type; and (5) type of technology mentioned. The analysis distinguished 3 main themes emerging from the literature: (1) impact and expectations of remote social contact on the physical and mental health and well-being of the residents (n=12), (2) with whom or what the social contact took place (n=17), and (3) limitations and barriers to significant social contact related to digital technologies (n=14). The results first underlined the highly positive impact expected by the authors of the digital technologies on health and quality of life of residents of long-term care facilities. Second, they highlighted the plurality of ties to consider, since social contact takes place not only with family caregivers to maintain contact but also for other purposes (end-of-life videoconferences) and with other types of contact (eg, with staff and robots). Third, they exposed the limitations and barriers to significant contact using digital technologies and outlined the required conditions to enable them.

**Conclusions:**

The review demonstrated the opportunities and risks outlined by the literature about the implementation of digital technologies to support remote social contact. It showed the plurality of ties to consider and revealed the need to evaluate the positive impact of remote contact from the residents’ perspectives. Therefore, to go beyond the risk of digital solutionism, there is a need for studies considering the holistic impact on health regarding the implementation of digital technologies, including the meaning residents give to interpersonal exchanges and the organizational constraints.

**Trial Registration:**

OSF Registries osf.io/yhpx3; https://osf.io/yhpx3

## Introduction

### Background

One of the most significant issues of the COVID-19 pandemic has been the effects on long-term care facility residents, who represent 50% of deaths in Europe [1]. Beyond the increase in mortality directly caused by COVID-19, the social distancing measures themselves accelerated declines in mental and physical health among some long-term care facility residents [2], as strict social isolation can cause psychological distress [3]; worsen depression, anxiety, and dementia; and contribute to failure to thrive [4]. In addition, there is some collateral damage caused by delayed surgery and dental care, which has been associated with depression issues [5].

Information and communication technology use has been considered a means to maintain older adults’ quality of life and provide them with solutions to fight the onset of depression, while also limiting face-to-face contact to protect them from the risks of viral transmission [6]. Beyond the impact on health and quality of life, this study considered the socialization needs of residents as important, considering socialization as a basic human need [7]. People in vulnerable situations require particular attention, especially when their ability to communicate their needs is altered, making such expression often difficult to comprehend. Therefore, we were particularly interested in the way digital devices have been implemented or envisaged meeting these socialization needs during visitation restrictions due to the pandemic.

Previous research has addressed the topic of long-term care facility residents’ remote social contact—social contact being defined as an exchange between 2 (or more) people [8]. Televisits with residents’ families were analyzed from the perspective of enhancing social presence or degree of salience and thus refer to the quality or state of being there when using a communication medium [9]. Televisits were compared with traditional telephone exchanges [10]. Videoconferencing with relatives has been reported to have a positive impact on social support, loneliness, and depressive status [11,12]. Social support is a “multi-dimensional construct, including emotional, appraisal, instrumental (or tangible), and informational support [...]. One important aspect of social support for older nursing home residents is the continued involvement of family members” [11]. Researchers have identified limitations such as inhibited videoconferencing use due to age-related cognitive decline and physical frailty [13] and the acceptability of videoconferencing by residents’ families, which is inversely proportional to the length of a resident’s stay [14].

A major contribution from work on this topic, which has primarily focused on cognitively intact residents, is that those most likely to use videoconferencing, considered the “second best option for visitation” [12], are those whose relatives live far away. During the COVID-19 pandemic, the problem of distance arose in another form, as even family members who lived nearby were not allowed to visit their relatives for a relatively long period. Each country and region implemented different restrictions, ranging from strict isolation in rooms to supervised visits during certain circumstances or with mitigating procedures. These restrictions have evolved over time, moving in some institutions from an initial absolute ban to adjusted visitation procedures as knowledge about the virus has evolved [15].

### Objective

Thus, this scoping review aimed to report on research articles that emerged during the period when the most stringent restrictions were in place, from March 2020 to June 2021. We focused on how technological devices have been or should be mobilized, according to the authors, to meet long-term care facility residents’ socialization needs. Although other recent scoping or rapid reviews addressing the pandemic context focused on social isolation among older adults [16], strategies and actions to enable residents to maintain meaningful family connections [17], or the impact of the pandemic on older adults [18], to our knowledge, our scoping review is the first to specifically address the socialization needs through digital means during visitation restrictions of long-term care facility residents. We therefore conducted a scoping review to provide an overview of existing research on the links between digital technologies and social isolation in long-term care facilities during the COVID-19 pandemic, guided by the following research question: How are the links between digital technologies and social isolation described in the current scientific literature for older adults living in nursing homes during the COVID-19 pandemic?

## Methods

### Protocol and Registration

This review was conducted according to the scoping review stages described by Arksey and O’Malley [19] and in the extension by Peters et al [20]. The steps include (1) formulating the research question; (2) identifying relevant studies; (3) selecting relevant studies; (4) charting the data; (5) collating, summarizing, and reporting the results; and (6) consultation. We used PRISMA-ScR (Preferred Reporting Items for Systematic Reviews and Meta-Analyses Extension for Scoping Reviews) [21]. The study was preregistered (see Multimedia Appendix 1 for details).

### Eligibility Criteria

Our eligibility criteria were developed within the PICo (Population or Problem, Interest, Context) framework (see Multimedia Appendix 2 for details). We included qualitative and quantitative studies, reviews, commentaries, viewpoints, and letters to the editors in French or English focusing on digital technologies aiming to supporting the social contact of residents in long-term care facilities during visitation restrictions due to the COVID-19 pandemic. A central criterion was that specific concepts are used to account for the social contact of residents (eg, social networks, social support) or lack thereof (eg, social isolation, loneliness). Even if the use of digital technologies in long-term care facilities was not the main topic, at least part of the article should be dedicated to it. We included articles published in peer-reviewed journals when the most stringent restrictions were in place, from March 2020 to June 2021.

We excluded articles in a language other than French and English and those in which we were unable to clearly identify the target population and context (eg, quantitative studies on older adults without specific focus on long-term care facility residents). We also excluded articles that did not focus on the older adult population and those with no clear correlation between technology use and visitation restrictions during the COVID-19 pandemic.

### Information Sources (Database Selection) and Search Strategy

Database selection was conducted in collaboration with librarians from the University of Strasbourg, who were specialists in this type of research. Our aim was to cover a broad disciplinary spectrum, combining research in the social sciences, humanities, management studies, economics, and health sciences. Thus, on June 29, 2021, we conducted research on 3 major platforms: EBSCO, ProQuest, and PubMed. Comprehensive searches were performed, combining keywords correlated with the following: (1) older adults, (2) their need for socialization, covered by (3) technological devices in (4) long-term care facilities during (5) the COVID-19 pandemic. First, we brainstormed to select terms related to these 5 themes, for example: (1) “Older people” OR “elderly” OR “aged.” Then, we searched each database to find related terms out of the Thesaurus (EBSCO and ProQuest) or MeSH Terms (PubMed). For an extensive overview of the search terms selected, please refer to Multimedia Appendix 3, and for a full overview of the searches conducted in each database, please refer to Multimedia Appendix 4. We limited the publication years at the beginning of 2020, that is, shortly before the time when stay-at-home orders emerged in most countries worldwide due to the pandemic, and included all articles meeting our criteria up to the day we conducted the search.

### Data Charting Process and Analysis

Several precautions were taken to limit selection bias. We separated the selected articles into 2 equal parts. All the titles were screened by CS and CH in the first subsection and by CL and CH in the second subsection. CL was responsible for arbitrating selection conflicts between CS and CH, and CS was responsible for arbitration between CL and CH. In the second stage, we followed a similar approach, this time focusing on the abstracts. All the titles were screened by CL and CH in the first subsection and by CS and CH in the second subsection. We adopted a 4-point scoring system, with a score of 1 being used to denote articles that were off-topic and a score of 4 for those that were fully aligned. To this, we added comments for the other authors. Papers scoring 4 twice, or at least 3 and 4, were immediately selected. Those below this score were excluded from the first round. We excluded works that did not specifically address the older adult population or clearly excluded long-term care facility residents (eg, “community-dwelling older adults” in the title or abstract). We also excluded articles written in a language other than French or English. CS was responsible for arbitrating selection conflicts between CL and CH, and CL was responsible for arbitration between CS and CH.

In the third step, we screened the full text of the retained articles, each of us responsible for one-quarter of the articles. Together, the study authors formalized a standardized data extraction sheet, in which each author recorded the following for the articles relevant to them: authors, scientific discipline, title, year and month of publication, country of origin, keywords, type of article (eg, view, review, qualitative research), purpose, methods, technology type, key findings (text quote), key concepts, variables used, practical implications, and research perspectives. A simplified version of this table is available in Multimedia Appendix 5.

## Results

### Selection of Sources of Evidence

**Figure 1:**
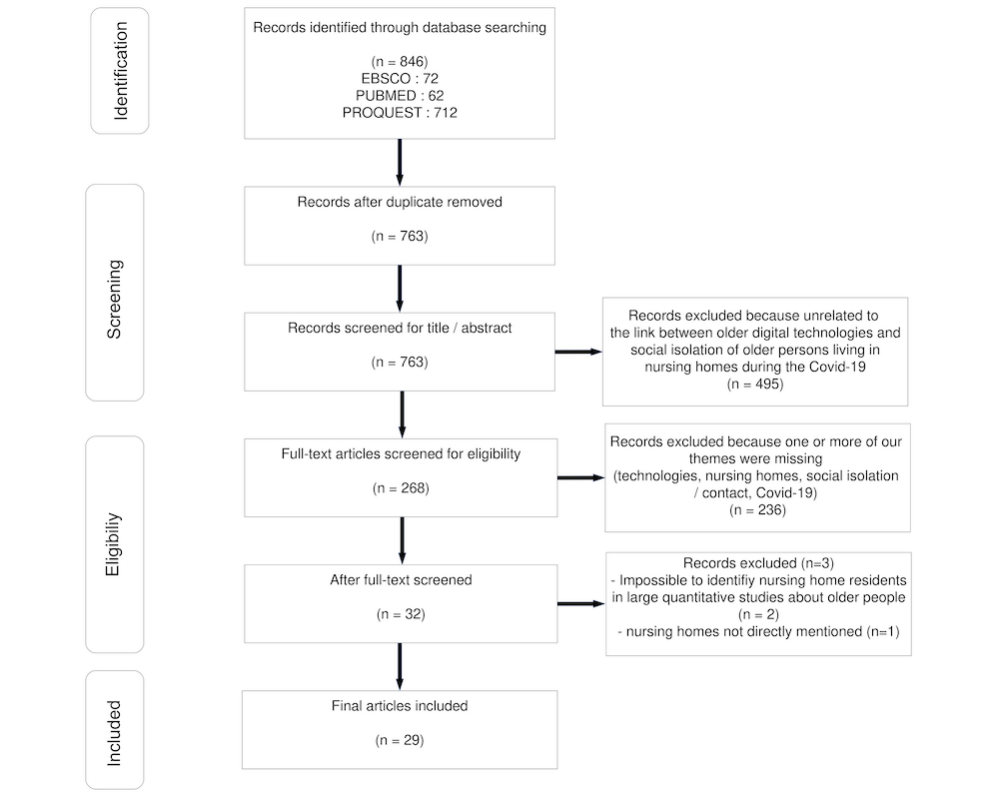
Flow diagram for the search and selection process.

Once duplicates were removed, we had retrieved 763 articles. These were mainly published by researchers or hospital practitioners with the aim of shedding light on the situation. Only articles from peer-reviewed journals were selected. Excluded articles were those in which resident socialization and use of digital devices were not directly correlated as a primary or secondary theme (in a subsection). However, we chose to include articles on past research if the authors discussed it in relation to the current pandemic context. This led us to select 268 articles that were screened for eligibility. We rejected 232 articles because one or more of our selected themes was missing. Finally, of the 32 articles selected at this stage, 3 more were removed: 2 were quantitative studies in which long-term care facility residents were included in a larger panel of older adults, without this population being analyzed specifically. Another viewpoint article was removed because long-term care facilities were not directly specified. We thus selected 29 articles for analysis (Figure 1).

### Characteristics of Articles Included in the Review

Multimedia Appendix 5 details the characteristics of the 29 articles: (1) authors, title, and date of publication; (2) country of the first author; (3) research fields; (4) article type; (5) type of technology mentioned.

Among the 29 included articles, 12 were published between March 2020 and July 2020, during the early months of the pandemic; 22 were published in 2020, and 7 were published between January 2021 and June 2021.

Regarding the first authors of the articles, 13 were from North America, 11 were from Europe, 2 were from Australia, 2 were from Asia, and 1 was from the Middle East. Of the articles, 4 were from an international perspective, 6 were multidisciplinary (crossing disciplines from health sciences, human and social sciences, and engineering), 18 were primarily from a health sciences perspective (nursing science, psychiatry, public health, psychogeriatric, geriatrics, medicine), 4 were written mainly by social science researchers (anthropology, social work, psychology), and 1 was written by a “think-and-do tank” director.

Regarding article type, 14 articles were commentaries (n=9), viewpoints (n=4), or a letter to the editor (n=1). There were 7 review articles, including systematic (n=1), narrative (n=2), scoping (n=2), protocol for a scoping (n=1), and rapid (n=1) reviews, and 8 articles were based on either qualitative (n=5) or quantitative (n=3) empirical studies.

We then conducted a qualitative analysis of the papers included to answer our research questions. It appeared heuristic to distinguish the articles according to the following: (1) impact and expectations of remote social contact on the physical and mental health and well-being of the residents (n=12), (2) with whom or what the social contact took place (n=17), and (3) limitations and barriers to significant social contact related to digital technologies (n=14).

### Expectations of Remote Social Contact on Residents’ Health and Well-being

The first expectation regarding digital technologies used in long-term care facilities during the visitation restrictions related to the COVID-19 pandemic was enablement of residents’ social contact because of its positive effects. To address the positive effects of social contact, the authors used several terms. Many articles referred to the residents’ socialization needs through the lack of social contact [7,16,22-24]. Authors used the concept of “social isolation” to describe the absence of face-to-face contact, which can be measured objectively [16]; it usually correlated with the concept of “loneliness,” which is the “subjective experience of feeling alone or disconnected from others” [25]. Other authors referred to the residents’ socialization needs through social connection [7,17,23,26-28]. Bethell et al [7] defined “social connection” through the combination of the following interrelated concepts: social networks (webs of social relationships), social support (emotional, social, physical, and financial help), social engagement (taking part in activities), and social connectedness (feelings of being cared for and belonging). Social integration was related to the belonging to a social network [29].

Most authors cited in this subsection assumed that technology use would necessarily have a positive effect. The use of technologies to communicate (eg, video calls via phones and tablets) was reported to promote social connection [7] and allow residents with dementia to engage well with others [24]. The impact of technology use on quality of life by facilitating communication with family members or for video consultations was also noted [22]. Social isolation could be alleviated by connecting with the outside world, gaining social support, boosting self-confidence, and engaging in activities of interest [30]. Social connections through technology were linked to enhanced well-being, protecting and improving mental health, and maintaining physical health and independence among older adults [18]. Office et al [31] noted the positive impact of a telephone befriending program on older adults’ perceived health and well-being. Providing technology-dependent amenities to long-term care facility residents could also increase their self-perceived health [32]. In their narrative review, Gorenko et al [25] cited a study demonstrating that video calls set up by staff could have positive effects on depression and loneliness.

However, some work did not address so much the positive effects of social contact at a distance but rather the prevention of the negative effects of social distance. Thus, the integration of digital connectivity could help overcome social isolation and loneliness [26] or at least reduce their prevalence [33]. Social isolation can lead to fear, depression, anxiety [34], cognitive decline, fatigue, and sleep disturbances [23]. This implies less infection resistance, more emergency admissions to the hospital, and extended lengths of stay [31]. Technologies were presented as a “key tool” for reducing social isolation [23] for long-term care facility residents who essentially became prisoners in their 1-bedroom living spaces, with this extreme loneliness potentially inducing anxiety, depression, malnourishment, and worsening dementia [32]. Technological devices have been presented as “a boon for residents feeling isolated” [34] regardless of the type: Videoconferencing has shown the same positive effects on depressive symptoms or loneliness regardless of whether a smartphone or a laptop is used [30]. For example, programs were developed for medical student volunteers to have weekly telephone calls with long-term care facility residents [16,31].

Telephone befriending programs can have bidirectional benefits, as students feel empowered by being able to make a difference in the lives of socially isolated seniors [31]. Thus, the authors of the selected articles not only addressed the effects of remote social contact on residents but also focused on their interlocutors, both human and nonhuman, as in the case of interactions with social robots.

### With Whom (or What) the Social Contact Takes Place

The selected articles paid particular attention to families, with whom it would be essential to maintain a connection. Information and communication technologies should mainly improve meaningful connections between older people and their families in long-term care facilities during the pandemic [17].

Initiatives have emerged worldwide to provide older adults with a connection to the “real world” and means for communicating with friends and family [26]. Public authorities have also addressed this issue. For example, the Italian Ministry of Health published a circular requiring residential facilities to provide residents with access to their families and friends through technological communication to facilitate social occasions and affective interactions [33].

Many interventions to maintain connections between residents and their families were addressed [27], such as the “Friend in Deed” program [28] or the Rhode Island assisted living facility program, which distributed tablets to residents to video call their families, thus facilitating social engagement [23]. Other “useful social contacts” included other care home residents, increasing their social networks by connecting two or more care homes through video calls over a long period, within the framework of a study [35].

There was a specific issue of concern in 3 articles included in this scoping review: the use of technology to avoid dying alone despite the impossibility of visits [27]. Videoconferencing was useful for family visits or consultations for patients dying from COVID-19 complications [16], providing a last chance for older adults and their relatives to “say goodbye” [17]. Two articles addressed the perspectives of residents’ relatives, showing that online communication provided them with support through their social networks [36] to cope with their loved one’s end of life and death. It is indeed possible to share online resources for bereavement support [28].

In addition to family relationships, care modalities (and, consequently, social contact with professionals) also evolved with technology during the pandemic, although not much discussion was provided on this issue. The topic was addressed by 2 articles, showing, for example, that telehealth solutions for geriatric mental health care [26], such as telephone or video conferences [34], allowed for the delivery of convenient, accessible, and affordable care [26]. Although rarely mentioned in the literature, staff members have also asked to use technological solutions to connect with residents (virtual therapy sessions, telehealth visits, video calls with friends and family, virtual activities in their rooms [16]). Special attention was also paid to families, as some social workers developed telesupport interventions for family caregivers [37].

From another perspective, 4 articles addressed the relationships between humans and technology, showing that direct contact with technological devices can help long-term care facility residents cope with social distancing related to COVID-19. Social robots can have a positive impact on loneliness by enhancing autonomy, increasing levels of engagement [38], and encouraging attachment and social integration among older adults [29]. For example, Sunshine’s robot’s (a Korean-manufactured, English-speaking doll-chatbot system) conversations (playing songs, cueing reminiscences, quoting inspirational passages, telling stories, playing “Simon Says”) could encourage exercise and social engagement and calm agitation; may have positive effects on geriatric depression, sleep quality, and cognition; and can encourage previously unresponsive residents to express themselves [39]. Further, direct social robots are considered a means to empower older adults with few social resources: Playing a mediating role in the care of older adults, they contribute to creating social connectivity [29]. Augmented reality was also noted as a way to reduce the burden of frailty and increase well-being and social participation [32].

However, the impact of direct social robots (eg, animal robots with various sensors that can react to stimuli or software humanoid agents that assess older adults’ affective states and engage in daily conversations with the aim of reducing social isolation) is nuanced [40]. They mainly provide emotional support, whereas online social platforms, for example, provide easy access to information sources and opportunities to communicate. Online social platforms are multifaceted systems that are expected to promote social participation, cognition, physical activity, nutrition, and sleep.

Authors do not always take for granted that technological solutions are easy to implement and correspond to residents’ needs. Some articles selected for this systematic scoping review also pointed out obstacles and limitations, which we address in the following subsection.

### Limitations and Barriers to Significant Social Contact Related to Digital Technologies

This review showed that neither the use of technology nor the establishment of real social ties is automatic. Implementing video communications requires an adequate organizational structure and consideration of the ability of the residents’ family members to use the technology [41]. Some authors have considered the digital exclusion of residents’ families [28,33] as well as infrastructural and staffing constraints in long-term care facilities [7,25,42-44]. Moyle et al [13] outlined the time needed to mediate videoconferencing and the difficulty of adding it to the team’s workload, which requires the long-term care facility teams to reorganize their functioning.

Among the 29 articles, 14 highlighted the barriers of using technology to meet long-term care facility residents’ social needs. This subsection discusses the limits of technology used to reduce isolation in general; however, the authors of the reviewed articles mainly focused on the digital divide and (the limits of) technological devices. Only Vernooij-Dassen et al [45] stated that “older adults need more than virtual contacts.”

The digital divide was approached in the reviewed studies from several angles. First, older adults often need assistance using digital technologies, and the most vulnerable have no access to web resources or the required digital skills [18]. Older age, combined with lower income and less education, can lead to reduced access to technology [26], inducing a notably negative impact on access to mental health care [46]. According to Eghtesadi [32], exclusion from technological advances may be due to negative representations of older adults (eg, passivity and lack of capacity to learn), combined with the fact that this population often cannot self-advocate. The digital divide concerns not only older adults but also their families [28], insofar as social disparities restrict technology access [33].

Some authors posited correlations between digital socialization and quality of life but noted that this will not systematically be efficient for residents with low digital literacy [22], especially those with cognitive impairments [22,26]. Fears regarding the security of personal data, difficulties in accessing dedicated tools, and visual or hearing impairments were also identified as barriers [16], which add to the fact that long-term care facility residents do not necessarily understand the interest of digital tools, as in the case of telehealth visits [47]. Several infrastructural issues and limitations have been highlighted, such as staff members’ availability to set up video calls, their access to these types of devices, their capacity to schedule and facilitate these interactions [42], and staff commitment and turnover [25]. Access to technology in long-term care facilities does not necessarily dictate its optimal use [43]. To have a real impact on social isolation and loneliness, professionals need to assist older adults use digital tools [44].

Several considerations should be taken into account: the purchase of dedicated equipment and infrastructure (eg, wireless networks), the allocation of dedicated professional time to accompany each resident, and the issue of human resource capacities (eg, staff training, volunteer recruitment [7].

## Discussion

### Principal Findings

Overall, this scoping review gives a comprehensive overview of the current literature and shows how the scientific papers published during the period of restricted visits due to the COVID-19 pandemic considered the contribution of digital technologies to residents’ social contact. We can summarize our main findings in 3 points. First, we outlined the main expectations for digital technologies to prevent social isolation and loneliness and defined the terms used. The positive impacts expected of remote connections are detailed as well as the negative effects prevented by the use of digital technologies. Second, while prepandemic work on the topic, as described in the Introduction section, mainly focused on the tie between residents and their families, our study shows that articles published during the COVID-19–related confinement and visitors’ restrictions focused on a plurality of ties. Indeed, social contact took place not only with family caregivers to maintain contact with the residents but also for other purposes (end-of-life videoconferences) and with other types of contact: Several articles addressed remote ties with professional caregivers, student volunteers, and residents of other institutions and even direct contact with social robots. Digital socialization thus concerns intrafamily ties and a broader network of ties. Third, we reported on the limitations and barriers to significant contact using digital technologies (digital divide and access difficulties, notably due to cognitive impairment and the low digital literacy of residents and their relatives) and outlined the required conditions to enable them, in particular organizational settings (technological infrastructure, dedicated professional time, human resource capacities).

### Comparison With Prior and Recent Work

In the literature, the expectations for digital technologies to ensure significant remote connections are high. Digital solutions are generally seen as a natural alternative to face-to-face contact (one exception is Vernooij-Dassen et al [45]). They are considered as suitable methods to care for chronically ill, frail, or dependent older adults while also reducing health care costs [48]. These devices could allow residents to have a new, interesting device to show to visitors, making them an object of social mediation [49] and contributing to the creation of social connectivity [28]. To avoid a tendency for technological solutionism [50], considering that the implementation and appropriation of technologies are synonyms, it seems essential to finely define the implications and entanglements and identify the contributions and limitations of digital solutions used to maintain or develop social ties. This aim can be achieved through an increased number of studies based on empirical methods [51]. Indeed, previous studies on long-term care facility residents’ remote social ties have used standardized scales, such as the Geriatric Depression Scale [10], combined with loneliness and social support behavior scales [11] or with loneliness and quality of life scales [30]. In the corpus studied in our review, some empirical articles evaluating the impact of remote social contact through the lens of health and quality of life issues were conducted before the COVID-19 crisis [29,30,38]. However, the pandemic context itself can be anxiety-provoking [52] and thus influence evaluations related to residents’ health, solitude, and quality of life. Studies conducted during the crises and questioning the significance of the connections are thus required. Moreover, expectations cannot be addressed in a uniform manner, as if residents all have the same identity and history. Multiple dimensions of social identity, such as gender, age, or migration status [53], need to be considered in the studies because they impact the use of digital technologies and meaning of social contact. Conversely, it is necessary to consider the barriers to remote social contact and virtual care, which increased following the pandemic, due to old age [54,55] combined with other characteristics like ethnicity [56].

Regarding the plurality of ties through remote social contact stated in our review, we found that most of the previous studies focused on the relationships between residents and their families [41]. Above all, our results show that extrafamilial ties need to be considered, not only because family involvement after long-term care facility admission can quantitatively decrease [14] but also because other ties are important. Indeed, some residents have remote contact with residents from other long-term care facilities [35], some professionals request digital contact with long-term care facility residents [16], and some meaningful exchanges occur via telehealth solutions [26,34]. Since the related practices are based on affects and moral feelings [57,58] for both caregivers and care receivers [59], we could consider all their social ties: the “family structure, the state and nature of their social relationships both inside and outside the nursing home, and their social practices” [60].

Concerning the barriers and enabling settings for significant remote social contact in long-term care facilities, consideration of organizational issues is novel in the COVID-19–related studies. Although past work did not focus on this subject [41], the organizational perspective of remote social contact mediation is addressed in various articles, particularly in terms of staffing constraints. The literature shows that, since an increase in social service and activity staff has a positive impact on residents’ quality of life [61], long-term care facilities could train their staff to mediate and implement remote social contact. This could be a way to better respond to crises, prepare for the future needs of the residents, and limit the turnover of professionals, as stated in previous work [62].

This review enables us to consider the opportunities for residents' literacy (highlighted as a barrier to significant remote social contact) by collecting and taking into account their requests for remote connection. The articles reviewed for this scoping review report on neither the residents’ needs for digital contact nor their lived experiences, even in the articles that referred to direct interventions [27,28,31]. Indeed, qualitative studies developed during the pandemic assessed the impact of volunteer phone calls on social isolation [31] or the effect of remote Quizz sessions between residents of several long-term care facilities [35] or stated the usefulness of the technologies used in the research [42]. Quantitative studies measured the number of long-term care facilities reporting the use of digital devices [23] or residents’ preferences between phone and video calls [63]. None of the studies considered the perspective of the residents, as previous research has stated. The need for well-developed and tested interventions was indeed highlighted by Palmdorf et al [51], who showed that there is a lack of information about the actual needs of the users of digital technologies.

### Limitations

Several limitations concerning our review need to be highlighted. First, the systematic search approach may have been biased, particularly because we were limited by the subscriptions to which our affiliated university provided us access. We cannot exclude the possibility that we overlooked some work published during the examined time period that was related to our research questions. In the future, this bias could be avoided by soliciting co-authors from other universities. Second, our inclusion criterion that only articles from peer-reviewed journals should be included potentially led us to not identify certain work, such as from the grey literature. A less restrictive scoping review, including this grey literature, could be conducted on the same basis. The third limitation is specifically related to our research topic and the period covered. It is very difficult to conduct research during large-scale crises, especially in long-term care facilities that have been particularly affected. Therefore, the empirical material collected by the authors of the reviewed articles is hardly representative or may even be nonexistent in most of the selected articles. A new scoping review using our approach could be conducted covering a longer period, assuming that some work may have appeared later.

### Future Directions

To capture the demand and need of the residents, more in-depth evaluations should be methodologically conducted. Social support, social network, social engagement, and social connectedness should be distinguished in the measurements. These approaches should be complemented by qualitative methodologies to outline residents’ subjective experiences regarding digital device use and elucidate the individual, interpersonal, and organizational specifics that impact the experience. Further, we should investigate the social support provided by the staff [42].

Future studies should situate their analyses in a temporal context (before, during, and after social distancing and visitor restrictions), as has been done by researchers with older adults living at home [64], and consider the organizational, geographical, and material dimensions in which the interactions take place. Taking into consideration the expectations of the individuals would also state the gap between the imagined future (generally idealized) and the actual appropriation of the devices [65]. Regarding direct contact with social robots, the *nature* of this type of tie needs could also be further questioned.

Research programs could be implemented on an international scale in the digital health field, considering the pandemic context, to provide solutions for maintaining and improving the living conditions of older adults in a broad sense, including those living in long-term care facilities [66].

Future studies should analyze if and how remote social contact allows families to stay effective care partners and not solely remote “visitors” during and after COVID-19 epidemic peaks [67]. In other words, it is a matter of evaluating whether these digital devices allow relatives to carry out the family’s care work “at a distance.”

### Conclusions

This review demonstrated the opportunities and risks outlined by the literature about the implementation of digital technologies to support remote social contact. If the expectations for digital technologies to support significant remote connections are high, the review showed that studies conducted during the crises and questioning the significance of the connections are thus required. This review also showed the plurality of ties to consider and revealed the need to evaluate the positive impact of remote contact from the resident’s perspective. Therefore, to go beyond the risk of digital solutionism, there is a need for studies considering the holistic impact of digital technology implementation on health, including the meaning residents give to interpersonal exchanges and the organizational constraints.

This scoping review opens up perspectives for policy makers in terms of political planning and for long-term care facility managers who have to implement these policies with their staff. Beyond the sole epidemic context, this review’s findings make it possible to identify points of vigilance for implementing digital devices dedicated to socialization among long-term care facility residents, in anticipation of the “digital revolution in health” [68] and care, in the context of demographic aging [69] and increased geographical mobility among new generations [70].

## Appendix

Multimedia Appendix 1 PRISMA ScR checklist.

Multimedia Appendix 2 Application of the Population or Problem, Interest, Context (PICo) framework.

Multimedia Appendix 3 Search terms.

Multimedia Appendix 4 Search equations.

Multimedia Appendix 5 Main characteristics of the selected articles.

## Abbreviations

PICo: Population or Problem, Interest, Context

## Data Availability

The data sets generated during or analyzed during the current study are available from the corresponding author on reasonable request.

## References

[1] Booth RG, Andrusyszyn M, Iwasiw C, Donelle L, Compeau D (2016). Actor-Network Theory as a sociotechnical lens to explore the relationship of nurses and technology in practice: methodological considerations for nursing research. Nurs Inq.

[2] Saldaris M (2021). The dichotomy of social isolation in a global pandemic: when the power to protect actually harms. Journal of Health Care Finance.

[3] Kim HS, Jung J (2021). Social isolation and psychological distress during the COVID-19 pandemic: a cross-national analysis. Gerontologist.

[4] Abbasi J (2020). Social isolation-the other COVID-19 threat in nursing homes. JAMA.

[5] Luo Y (2021). The association of delayed care with depression among US middle-aged and older adults during the COVID-19 pandemic: cross-sectional analysis. JMIR Aging.

[6] Csesznek C, Cersosimo G, Landolfi L (2020). New challenges for the elderly: a sociological reflection on socialization to ICT's as an opportunity in the time of the Covid-19. Revista Romana de Sociologie.

[7] Bethell J, O'Rourke HM, Eagleson H, Gaetano D, Hykaway W, McAiney C (2021). Social connection is essential in long-term care homes: considerations during COVID-19 and beyond. Can Geriatr J.

[8] Mossong J, Hens N, Jit M, Beutels P, Auranen K, Mikolajczyk R, Massari M, Salmaso S, Tomba GS, Wallinga J, Heijne J, Sadkowska-Todys M, Rosinska M, Edmunds WJ (2008). Social contacts and mixing patterns relevant to the spread of infectious diseases. PLoS Med.

[9] Parker EB, Short J, Williams E, Christie B (1976). The Social Psychology of Telecommunications.

[10] Mickus MA, Luz CC (2002). Televisits: sustaining long distance family relationships among institutionalized elders through technology. Aging Ment Health.

[11] Tsai H, Tsai Y (2011). Changes in depressive symptoms, social support, and loneliness over 1 year after a minimum 3-month videoconference program for older nursing home residents. J Med Internet Res.

[12] Tsai H, Tsai Y, Wang H, Chang Y, Chu HH (2010). Videoconference program enhances social support, loneliness, and depressive status of elderly nursing home residents. Aging Ment Health.

[13] Moyle W, Jones C, Murfield J, Liu F (2020). 'For me at 90, it's going to be difficult': feasibility of using iPad video-conferencing with older adults in long-term aged care. Aging Ment Health.

[14] Tsai H, Tsai Y (2015). Attitudes toward and predictors of videoconferencing use among frequent family visitors to nursing home residents in Taiwan. Telemed J E Health.

[15] Hugelius K, Harada N, Marutani M (2021). Consequences of visiting restrictions during the COVID-19 pandemic: An integrative review. Int J Nurs Stud.

[16] MacLeod S, Tkatch R, Kraemer S, Fellows A, McGinn M, Schaeffer J, Yeh CS (2021). COVID-19 era social isolation among older adults. Geriatrics (Basel).

[17] Veiga-Seijo R, Miranda-Duro MDC, Veiga-Seijo S (2022). Strategies and actions to enable meaningful family connections in nursing homes during the COVID-19: a scoping review. Clin Gerontol.

[18] Lebrasseur A, Fortin-Bédard N, Lettre J, Raymond E, Bussières EL, Lapierre N, Faieta J, Vincent C, Duchesne L, Ouellet M, Gagnon E, Tourigny A, Lamontagne M, Routhier F (2021). Impact of the COVID-19 pandemic on older adults: rapid review. JMIR Aging.

[19] Arksey H, O'Malley L (2005). Scoping studies: towards a methodological framework. International Journal of Social Research Methodology.

[20] Peters MDJ, Godfrey CM, Khalil H, McInerney P, Parker D, Soares CB (2015). Guidance for conducting systematic scoping reviews. Int J Evid Based Healthc.

[21] Tricco Andrea C, Lillie Erin, Zarin Wasifa, O'Brien Kelly K, Colquhoun Heather, Levac Danielle, Moher David, Peters Micah D J, Horsley Tanya, Weeks Laura, Hempel Susanne, Akl Elie A, Chang Christine, McGowan Jessie, Stewart Lesley, Hartling Lisa, Aldcroft Adrian, Wilson Michael G, Garritty Chantelle, Lewin Simon, Godfrey Christina M, Macdonald Marilyn T, Langlois Etienne V, Soares-Weiser Karla, Moriarty Jo, Clifford Tammy, Tunçalp Özge, Straus Sharon E (2018). PRISMA Extension for Scoping Reviews (PRISMA-ScR): Checklist and Explanation. Ann Intern Med.

[22] Ayalon L, Zisberg A, Cohn-Schwartz E, Cohen-Mansfield J, Perel-Levin S, Bar-Asher Siegal E (2020). Long-term care settings in the times of COVID-19: challenges and future directions. Int. Psychogeriatr.

[23] Gallo Marin B, Wasserman P, Cotoia J, Singh M, Tarnavska V, Gershon L, Lester I, Merritt R (2020). Experiences of Rhode Island assisted living facilities in connecting residents with families through technology during the COVID-19 pandemic. R I Med J (2013).

[24] Gorenko JA, Konnert C, Speirs C (2021). Does caregiving influence planning for future aging?: a mixed methods study among caregivers in Canada. Res Aging.

[25] Gorenko JA, Moran C, Flynn M, Dobson K, Konnert C (2021). Social isolation and psychological distress among older adults related to COVID-19: a narrative review of remotely-delivered interventions and recommendations. J Appl Gerontol.

[26] Sano M, Lapid MI, Ikeda M, Mateos R, Wang H, Reichman WE (2020). Psychogeriatrics in a world with COVID-19. Int. Psychogeriatr.

[27] Lapid MI, Koopmans R, Sampson EL, Van den Block L, Peisah C (2020). Providing quality end-of-life care to older people in the era of COVID-19: perspectives from five countries. Int. Psychogeriatr.

[28] Burke S (2020). Stronger together? Intergenerational connection and Covid-19. QAOA.

[29] Pirhonen J, Tiilikainen E, Pekkarinen S, Lemivaara M, Melkas H (2020). Can robots tackle late-life loneliness? Scanning of future opportunities and challenges in assisted living facilities. Futures.

[30] Tsai H, Cheng C, Shieh W (2020). Effectiveness of laptop-based versus smartphone-based videoconferencing interaction on loneliness, depression and social support in nursing home residents: A secondary data analysis. J Telemed Telecare.

[31] Office EE, Rodenstein MS, Merchant TS, Pendergrast TR, Lindquist LA (2020). Reducing social isolation of seniors during COVID-19 through medical student telephone contact. J Am Med Dir Assoc.

[32] Eghtesadi M (2020). Breaking social isolation amidst COVID-19: a viewpoint on improving access to technology in long-term care facilities. J Am Geriatr Soc.

[33] Bolcato M, Trabucco Aurilio M, Di Mizio G, Piccioni A, Feola A, Bonsignore A, Tettamanti C, Ciliberti R, Rodriguez D, Aprile A (2021). The difficult balance between ensuring the right of nursing home residents to communication and their safety. Int J Environ Res Public Health.

[34] Pachana NA, Beattie E, Byrne GJ, Brodaty H (2020). COVID-19 and psychogeriatrics: the view from Australia. Int. Psychogeriatr.

[35] Zamir S, Hennessy C, Taylor A, Jones R (2020). Intergroup 'Skype' quiz sessions in care homes to reduce loneliness and social isolation in older people. Geriatrics (Basel).

[36] Moore KJ, Sampson EL, Kupeli N, Davies N (2020). Supporting families in end-of-life care and bereavement in the COVID-19 era. Int. Psychogeriatr.

[37] Lightfoot E, Moone RP (2020). Caregiving in times of uncertainty: helping adult children of aging parents find support during the COVID-19 outbreak. J Gerontol Soc Work.

[38] Casey D, Barrett E, Kovacic T, Sancarlo D, Ricciardi F, Murphy K, Koumpis A, Santorelli A, Gallagher N, Whelan S (2020). The perceptions of people with dementia and key stakeholders regarding the use and impact of the social robot MARIO. Int J Environ Res Public Health.

[39] Lee OE, Davis B (2020). Adapting 'Sunshine,' a socially assistive chat robot for older adults with cognitive impairment: a pilot study. J Gerontol Soc Work.

[40] Choi HK, Lee SH (2021). Trends and effectiveness of ICT interventions for the elderly to reduce loneliness: a systematic review. Healthcare (Basel).

[41] Schuster AM, Hunter EG (2019). Video communication with cognitively intact nursing home residents: a scoping review. J Appl Gerontol.

[42] Freidus A, Shenk D, Wolf C (2020). A rapid qualitative appraisal of the impact of COVID-19 on long-term care communities in the United States: perspectives from area aging staff and advocates. Human Organization.

[43] Luscombe N, Morgan-Trimmer S, Savage S, Allan L (2021). Digital technologies to support people living with dementia in the care home setting to engage in meaningful occupations: protocol for a scoping review. Syst Rev.

[44] Sacco G, Lléonart S, Simon R, Noublanche F, Annweiler C, TOVID Study Group (2020). Communication technology preferences of hospitalized and institutionalized frail older adults during COVID-19 confinement: cross-sectional survey study. JMIR Mhealth Uhealth.

[45] Vernooij-Dassen M, Verhey F, Lapid M (2020). The risks of social distancing for older adults: a call to balance. Int. Psychogeriatr.

[46] Chong TWH, Curran E, Ames D, Lautenschlager NT, Castle DJ (2020). Mental health of older adults during the COVID-19 pandemic: lessons from history to guide our future. Int. Psychogeriatr.

[47] Marsh JL, O'Mallon M, Stockdale S, Potter DR (2020). Caring for vulnerable populations during a pandemic: literature review. International Journal of Caring Sciences.

[48] Oudshoorn N (2011). Telecare technologies and the transformation of healthcare.

[49] Pols J, Moser I (2009). Cold technologies versus warm care? On affective and social relations with and through care technologies. Alter.

[50] Morozov E (2013). To Save Everything, Click Here: The Folly of Technological Solutionism.

[51] Palmdorf S, Stark AL, Nadolny S, Eliaß G, Karlheim C, Kreisel SH, Gruschka T, Trompetter E, Dockweiler C (2021). Technology-assisted home care for people with dementia and their relatives: scoping review. JMIR Aging.

[52] Bergman YS, Cohen-Fridel S, Shrira A, Bodner E, Palgi Y (2020). COVID-19 health worries and anxiety symptoms among older adults: the moderating role of ageism. Int. Psychogeriatr.

[53] Guruge S, Sidani S, Wang L, Sethi B, Spitzer D, Walton-Roberts M, Hyman I (2019). Understanding social network and support for older immigrants in Ontario, Canada: protocol for a mixed-methods study. JMIR Aging.

[54] Pak R, Price MM, Thatcher J (2009). Age-sensitive design of online health information: comparative usability study. J Med Internet Res.

[55] Kunonga TP, Spiers GF, Beyer FR, Hanratty B, Boulton E, Hall A, Bower P, Todd C, Craig D (2021). Effects of digital technologies on older people's access to health and social care: umbrella review. J Med Internet Res.

[56] Pham Q, El-Dassouki N, Lohani R, Jebanesan A, Young K (2022). The future of virtual care for older ethnic adults beyond the COVID-19 pandemic. J Med Internet Res.

[57] Branicki LJ (2020). COVID-19, ethics of care and feminist crisis management. Gend Work Organ.

[58] Gilligan C (1982). In a Different Voice: Psychological Theory and Women’s Development.

[59] Tronto JC (1993). Moral Boundaries: A Political Argument for an Ethic of Care.

[60] Bobillier Chaumon M, Michel C, Tarpin Bernard F, Croisile B (2013). Can ICT improve the quality of life of elderly adults living in residential home care units? From actual impacts to hidden artefacts. Behaviour & Information Technology.

[61] Bowblis JR, Roberts AR (2020). Cost-effective adjustments to nursing home staffing to improve quality. Med Care Res Rev.

[62] Castle NG, Engberg J, Anderson RA (2007). Job satisfaction of nursing home administrators and turnover. Med Care Res Rev.

[63] Sacco G, Lléonart Sébastien, Simon R, Noublanche F, Annweiler C, TOVID Study Group (2020). Communication Technology Preferences of Hospitalized and Institutionalized Frail Older Adults During COVID-19 Confinement: Cross-Sectional Survey Study. JMIR Mhealth Uhealth.

[64] Kim S (2021). Exploring how older adults use a smart speaker-based voice assistant in their first interactions: qualitative study. JMIR Mhealth Uhealth.

[65] Brown N, Michael M (2003). A sociology of expectations: retrospecting prospects and prospecting retrospects. Technology Analysis & Strategic Management.

[66] Rylett RJ, Alary F, Goldberg J, Rogers S, Versteegh P (2020). Covid-19 and priorities for research in aging. Can J Aging.

[67] Kemp C (2021). #MoreThanAVisitor: families as "essential" care partners during COVID-19. Gerontologist.

[68] Beranger J, Rizoulieres R (2021). The Digital Revolution in Health.

[69] Kinsella K (2016). Demographic dimensions of global aging. Journal of Family Issues.

[70] Michielin F, Mulder CH, Zorlu A (2008). Distance to parents and geographical mobility. Popul. Space Place.

