# Supplementary material for: Use of Digital Technologies to Maintain Older Adults’ Social Ties During Visitation Restrictions in Long-Term Care Facilities: Scoping Review
Source: JMIR Aging. 2023 Feb 10;6:e38593. doi: 10.2196/38593 (PMC9924058; doi:10.2196/38593)
Supplement: Multimedia Appendix 1 [file aging_v6i1e38593_app1.doc]

| **Section** | **Item** | **PRISMA-ScR Checklist Item** | **Consideration of the item in the article** |
| --- | --- | --- | --- |
| **Title** | 1 | Identify the report as a scoping review. | Our article’s title is: Use of Digital Technologies for Older Adults’ Ties during Visitation Restrictions in Long-term Care Facilities: A Scoping Review |
| **Abstract** Structured summary | 2 | Provide a structured summary that includes (as applicable) background, objectives, eligibility criteria, sources of evidence, charting methods, results, and conclusions that relate to the review questions and objectives | Adapted to JMIR Aging. All items are included:  Background/Objective/Methods: includes eligibility criteria and charting methods/Results/Conclusion |
| **Introduction** | 3 | Describe the rationale for the review in the context of what is already known. Explain why the review questions/objectives lend themselves to a scoping review approach. | Done in “Background” part |
| Rationale |
| Objectives | 4 | Provide an explicit statement of the questions and objectives being addressed with reference to their key elements (e.g., population or participants, concepts, and context) or other relevant key elements used to conceptualize the review questions and/or objectives | Done in “Objective” part |
| **Methods** | 5 | Indicate whether a review protocol exists; state if and where it can be accessed (e.g., a Web address); and if available, provide registration information, including the registration number | Done in “Protocol and registration” part |
| Protocol and registration |
| Eligibility criteria | 6 | Specify characteristics of the sources of evidence used as eligibility criteria (e.g., years considered, language, and publication status), and provide a rationale. | Done in “Eligibility criteria” part |
| Information sources* | 7 | Describe all information sources in the search (e.g., databases with dates of coverage and contact with authors to identify additional sources), as well as the date the most recent search was executed. | Done in “Information sources (Database Selection) and Search Strategy” part |
| Search | 8 | Present the full electronic search strategy for at least 1 database, including any limits used, such that it could be repeated. | Done in “Information sources (Database Selection) and Search Strategy” part + Complete search strategy available in [Multimedia Appendix C] |
| Selection of sources of evidence† | 9 | State the process for selecting sources of evidence (i.e., screening and eligibility) included in the scoping review. | Done in “Information sources (Database Selection) and Search Strategy” part |
| Data charting process‡ | 10 | Describe the methods of charting data from the included sources of evidence (e.g., calibrated forms or forms that have been tested by the team before their use, and whether data charting was done independently or in duplicate) and any processes for obtaining and confirming data from investigators. | Done in “Data Charting Process and Analysis” part |
| Data items | 11 | List and define all variables for which data were sought and any assumptions and simplifications made. | Done in “Data Charting Process and Analysis” part |
| Critical appraisal of individual sources of evidence§ | 12 | If done, provide a rationale for conducting a critical appraisal of included sources of evidence; describe the methods used and how this information was used in any data synthesis (if appropriate). | Not applicable to our Scoping Review |
| Summary measures | 13 | Not applicable for scoping reviews. |  |
| Synthesis of results | 14 | Describe the methods of handling and summarizing the data that were charted. | Done in “Data Charting Process and Analysis” part |
| Risk of bias across studies | 15 | Not applicable for scoping reviews. |  |
| Additional analyses | 16 | Not applicable for scoping reviews. |  |
| **Results** | 17 | Give numbers of sources of evidence screened, assessed for eligibility, and included in the review, with reasons for exclusions at each stage, ideally using a flow diagram. | Done in “Selection of sources of evidence” part |
| Selection of sources of evidence |
| Characteristics of sources of evidence | 18 | For each source of evidence, present characteristics for which data were charted and provide the citations | Done in “Characteristics of Articles Included in the Review” part + Main characteristics of the selected articles in [Multimedia Appendix D] |
| Critical appraisal within sources of evidence | 19 | If done, present data on critical appraisal of included sources of evidence (see item 12). | Not applicable to our Scoping Review |
| Results of individual sources of evidence | 20 | For each included source of evidence, present the relevant data that were charted that relate to the review questions and objectives. | Done in “Characteristics of Articles Included in the Review” part + Main characteristics of the selected articles in [Multimedia Appendix D] |
| Synthesis of results | 21 | Summarize and/or present the charting results as they relate to the review questions and objectives | Done in “Expectations of remote social contact on residents’ health and well-being” + “With whom (or what) the social contact takes place” + “Limitations and barriers to significant social contact related to digital technologies” parts |
| Risk of bias across studies | 22 | Not applicable for scoping reviews. |  |
| Additional analyses | 23 | Not applicable for scoping reviews. |  |
| **Discussion** | 24 | Summarize the main results (including an overview of concepts, themes, and types of evidence available), link to the review questions and objectives, and consider the relevance to key groups | Done in “Principal Findings” and “Comparison to Prior and Recent Work” parts |
| Summary of evidence |
| Limitations | 25 | Discuss the limitations of the scoping review process. | Done in “Limitations” part |
| Conclusions | 26 | Provide a general interpretation of the results with respect to the review questions and objectives, as well as potential implications and/or next steps. | Done in “Conclusions” part |
| **Funding** | 27 | Describe sources of funding for the included sources of evidence, as well as sources of funding for the scoping review. Describe the role of the funders of the scoping review. | Done in “Acknowledgements” part |

Adapted from Tricco AC, Lillie E, Zarin W, et al. PRISMA Extension for Scoping Reviews (PRISMA-ScR): Checklist and Explanation. *Ann Intern Med*. 2018;169(7):467-473. doi:[10.7326/M18-0850](https://doi.org/10.7326/M18-0850)
