# Supplementary material for: Use of Digital Technologies to Maintain Older Adults’ Social Ties During Visitation Restrictions in Long-Term Care Facilities: Scoping Review
Source: JMIR Aging. 2023 Feb 10;6:e38593. doi: 10.2196/38593 (PMC9924058; doi:10.2196/38593)
Supplement: Multimedia Appendix 2 [file aging_v6i1e38593_app2.doc]

| PICo Framework | | |
| --- | --- | --- |
| **P:** Population or Problem | **I:** Interest | **Co:** Context |
|  |  |  |
| What are the characteristics of the **p**opulation or the patient? | **I**nterest relates to a defined event, activity, experience or process | **Co**ntext is the setting or distinct characteristics |
| Older adults | Digital technologies used to maintain social contact (activity) | In long-term care facilities during the COVID-19 pandemic |
