# Supplementary material for: Use of Digital Technologies to Maintain Older Adults’ Social Ties During Visitation Restrictions in Long-Term Care Facilities: Scoping Review
Source: JMIR Aging. 2023 Feb 10;6:e38593. doi: 10.2196/38593 (PMC9924058; doi:10.2196/38593)
Supplement: Multimedia Appendix 3 [file aging_v6i1e38593_app3.doc]

In red : terms added after consulting Thesaurus (EBSCO + PROQUEST)

In green : terms added after consulting MeSH terms (PUBMED)

| Population (title / abstract / keywords) | “Older people” OR “elderly” OR “aged” OR “aging” OR “ageing” OR “geriatric” OR “acilitygy*” OR “senior” or “older adults” |
| --- | --- |
|  |  |
| Interest (all fields) | “Social network*” or “social ties” or “ social engagement” or “social disengagement” or “social support” or “social isolation” or “social capital” or “social belonging” or “social connectedness” or “social participation” or “social contact” or “social activit*” or “social link” or “social presence” or “social fabric” or “social integration” or “social cohesion” or “community bond*” or “social framework” or “loneliness” or “social identification” or “social distanc*” or “social interaction” |
| Interest (title / abstract / keywords) | Technolog* OR ICT OR Telehealth OR Telecare OR Gerontechnolog* OR “Smart technolog*” OR “Assistive technolog*” OR “ambient-assisted living technolog*” or “information & communication technolog*” or “digital technolog*” or ehealth or telemedecin* |
| Context (title / abstract / keywords) | “Long-term care” OR “nursing home” OR “Care home” OR “assisted-living acility*” OR “Hospice home” or “Nursing care Facility*” or “aged care Facility*” |
| Context (all fields) | “covid-19” OR “2019-ncov” OR “sars-cov-2” OR “cov-19” OR lockdown OR quarantine OR shutdown OR “stay-at-home order” OR “Patient Isolation” |
