# Supplementary material for: Use of Digital Technologies to Maintain Older Adults’ Social Ties During Visitation Restrictions in Long-Term Care Facilities: Scoping Review
Source: JMIR Aging. 2023 Feb 10;6:e38593. doi: 10.2196/38593 (PMC9924058; doi:10.2196/38593)
Supplement: Multimedia Appendix 4 [file aging_v6i1e38593_app4.doc]

PROQUEST search equation (N=712)

| S1 | (((ti("Older people" OR "elderly"OR "aged" OR "aging" OR"ageing" OR "geriatric" OR"gerontolog*" OR "senior" OR"older adults") OR ab("Older people" OR "elderly" OR "aged" OR "aging" OR "ageing" OR "geriatric" OR "gerontolog*" OR "senior" OR "older adults") OR mainsubject("Older people" OR "elderly" OR "aged" OR "aging"  OR "ageing" OR "geriatric" OR "gerontolog*" OR "senior" OR "older adults")) AND PEER(yes)) AND (("Long-term care" OR "nursing home" OR "Care home" OR "assistedliving facilit*" OR "Hospice home" OR "Nursing care facilit*" OR "aged care facilit*") AND PEER(yes)) AND (("Social network*" OR "social ties" OR " social engagement" OR "social disengagement" OR "social support" OR "social isolation" OR "social capital" OR "social belonging" OR "social connectedness" OR "social participation" OR "social contact" OR "social activit*" OR "social link" OR "social presence" OR "social fabric" OR "social integration" OR "social cohesion" OR "community bond*" OR "social framework" OR "loneliness" OR "social identification" OR "social distanc*" OR "social interaction") AND PEER(yes))) AND PEER(yes) AND la.exact("English" OR "French") AND PEER(yes) AND pd(>20191231) |
| --- | --- |
| S2 | (Technolog* OR ICT OR Telehealth OR Telecare OR Gerontechnolog* OR "Smart  technolog*" OR "Assistive technolog*" OR "ambientassisted living technolog*" OR  "information & communication technolog*" OR "digital technolog*" OR ehealth OR  telemedecin*) AND PEER(yes) AND la.exact("English" OR "French") AND PEER(yes) AND pd(>20191231) |
| S3 | S1 AND S2 |
| Restriction Operators | Peer reviewed / Articles in English and French only / Articles published after 12312019 |
| Selected Datbases | Coronavirus Research Database, ERIC, Periodicals Archive Online, PTSDpubs, Publicly Available Content Database, Sociological Abstracts, Sociology Database |

EBSCO search equation (N=72)

| S1 | TX "Social network*" or "social ties" or " social engagement" or "social disengagement" or "social support" or "social isolation" or "social capital" or "social belonging" or "social connectedness" or "social participation" or "social contact" or "social activit*" or  "social link" or "social presence" or "social fabric" or "social integration" or "social cohesion" or "community bond*" or "social framework" or "loneliness" or "social identification" or "social distanc*" or "social interaction" |
| --- | --- |
| S2 | TX "Long-term care" OR "nursing home" OR "Care home" OR "assisted-living facilit*" OR "Hospice home" or "Nursing care facilit*" or "aged care facilit*" |
| S3 | TI ( "Older people" OR "elderly" OR "aged" OR "aging" OR "ageing" OR "geriatric" OR "gerontolog*" OR "senior" or "older adults" ) OR AB ("Older people" OR "elderly" OR "aged" OR "aging" OR "ageing" OR "geriatric" OR "gerontolog*" OR "senior" or "older adults" ) OR SU ("Older people" OR "elderly" OR "aged" OR "aging" OR "ageing" OR "geriatric" OR "gerontolog*" OR "senior" or "older adults" ) |
| S4 | TX Technolog* OR ICT OR Telehealth OR Telecare OR Gerontechnolog* OR "Smart technolog*" OR "Assistive technolog*" OR "ambient-assisted living technolog*" OR "information & communication technolog*" or "digital technolog*" or ehealth or telemedecin* |
| S5 | S1 AND S2 AND S3 AND S4 |
| Restriction Operators | Publication date: 01012020 → 12312022 / search also in the whole text of the articles / apply equivalent subjects / find all my search terms |
| Selected Databases (10) | Business Source Premier / EconLit / Humanities International Complete / Library, Information Science & Technology Abstract / Philosophers Index / Regional Business News / SocINDEX with Full Text / Vente et Gestion / Political Science Complete / World Politices Review |

PUBMED search equation (N=62)

| S1 | "Social network*" or "social ties" or " social engagement" or "social disengagement" or "social support" or "social isolation" or "social capital" or "social belonging" or "social connectedness" or "social participation" or "social contact" or "social activit*" or "social link" or "social presence" or "social fabric" or "social integration" or "social cohesion" or "community bond*" or "social framework" or "loneliness" or "social identification" or "social distanc*" or "social interaction" |
| --- | --- |
| S2 | "Long-term care" OR "nursing home" OR "Care home" OR "assisted-living facilit*" OR "Hospice home" or "Nursing care facilit*" or "aged care facilit*" |
| S3 | [Title/Abstract] OR [MeSH Terms]"Older people" OR "elderly" OR "aged" OR "aging" OR "ageing" OR "geriatric" OR "gerontolog*" OR "senior" or "older adults" |
| S4 | Technolog* OR ICT OR Telehealth OR Telecare OR Gerontechnolog* OR "Smart technolog*" OR "Assistive technolog*" OR "ambient-assisted living technolog*" or "information & communication technolog*" or "digital technolog*" or ehealth or telemedecin* |
| S5 | (#1) AND (#2) AND (#3) AND (#4) |
| Restrictions | From 2020-2021 |
