# Supplementary material for: Use of Digital Technologies to Maintain Older Adults’ Social Ties During Visitation Restrictions in Long-Term Care Facilities: Scoping Review
Source: JMIR Aging. 2023 Feb 10;6:e38593. doi: 10.2196/38593 (PMC9924058; doi:10.2196/38593)
Supplement: Multimedia Appendix 5 [file aging_v6i1e38593_app5.doc]

| **Authors / Title / Date of publication** | **Country of the first author** | **Research Fields** | **Article type** | **Type of technology mentioned** |
| --- | --- | --- | --- | --- |
|  |  |  |  |  |
| Ayalon, L., Zisberg, A., Cohn-Schwartz, E., Cohen-Mansfield, J., Perel-Levin, S., & Bar-Asher Siegal, E., Long-term care settings in the times of COVID-19: Challenges and future directions (July 2020). | Israel | Multidisciplinary | Commentary | Videocalling and video consultations |
| Bethell, J., O’Rourke, H. M., Eagleson, H., Gaetano, D., Hykaway, W., & McAiney, C., Social connection is essential in long-term care homes: considerations during COVID-19 and beyond (June 2020). | Canada | Multidisciplinary | Viewpoint | Phone and tablets (mainly) |
| Bolcato, M., Trabucco Aurilio, M., Di Mizio, G., Piccioni, A., Feola, A., Bonsignore, A., Tettamanti, C., Ciliberti, R., Rodriguez, D., & Aprile, A., The difficult balance between ensuring the right of nursing home residents to communication and their safety (January 2021). | Italy | Health Sciences | Commentary | All technological communication tools (telephone, videocalls, and email) |
| Burke, S., Stronger together? Intergenerational connection and Covid-19 (November 2020). | U.K. | Think and Do Tank Director | Viewpoint | Videocalling (mainly) |
| Casey, D., Barrett, E., Kovacic, T., Sancarlo, D., Ricciardi, F., Murphy, K., Koumpis, A., Santorelli, A., Gallagher, N., & Whelan, S., The perceptions of people with dementia and key stakeholders regarding the use and impact of the social robot MARIO (October 2020). | Ireland | Multidisciplinary | Qualitative study | Social robot, Mario |
| Choi, H. K., Lee, S. H., Trends and effectiveness of ICT interventions for the elderly to reduce loneliness: A systematic review (March 2021). | Korea | Health Sciences | Systematic review | Robots, platforms, tablets |
| Chong, T. W. H., Curran, E., Ames, D., Lautenschlager, N. T., & Castle, D. J., Mental health of older adults during the COVID-19 pandemic: Lessons from history to guide our future (April 2020). | Australia | Health Sciences | Viewpoint | -Technology to facilitate contact  -Telehealth |
| Eghtesadi, M., Breaking social isolation amidst COVID-19: A viewpoint on improving access to technology in long-term care facilities (May 2020). | Canada | Health Sciences | Viewpoint | Smartphones / tablets / virtual reality |
| Freidus, A., Shenk, D., & Wolf, C., A rapid qualitative appraisal of the impact of COVID-19 on long-term care communities in the United States: Perspectives from area aging staff and advocates (December 2020). | U.S.A. | Social Sciences | Qualitative research article | Facetime (or similar calls) |
| Gallo Marin, B., Wasserman, P., Cotoia, J., Singh, M., Tarnavska, V., Gershon, L., Lester, I., & Merritt, R., Experiences of Rhode Island assisted living facilities in connecting residents with families through technology during the COVID-19 pandemic (October 2020). | U.S.A. | Health Sciences | Quantitative research article | Amazon Fire tablets and Apple iPads. |
| Gorenko, J. A., Moran, C., Flynn, M., Dobson, K., & Konnert, C., Social isolation and psychological distress among older adults related to COVID-19: A narrative review of remotely-delivered interventions and recommendations (January 2021). | Canada | Social Sciences | Narrative review | Computers, tablets, smartphones, applications for videocalls |
| Lapid, M. I., Koopmans, R., Sampson, E. L., Van den Block, L., & Peisah, C., Providing quality end-of-life care to older people in the era of COVID-19: Perspectives from five countries (May 2020). | U.S.A. | Health Sciences | Commentary | Video-conferencing / Telemedicine |
| Lebrasseur, A., Fortin-Bédard, N., Lettre, J., Raymond, E., Eve-Line Bussières, Lapierre, N., Faieta, J., Vincent, C., Duchesne, L., Ouellet, M.-C., Gagnon, E., Tourigny, A., Lamontagne, M.-È., & Routhier, F., Impact of the COVID-19 pandemic on older adults: Rapid review (April 2021) | Canada | Multidisciplinary | Rapid review | Information and communication technologies |
| Lee, O. E., & Davis, B., Adapting ‘Sunshine,’ a socially assistive chat robot for older adults with cognitive impairment: A pilot study (July 2020). | U.S.A. | Multidisciplinary | Letter to the editor | Robot |
| Lightfoot, E., & Moone, R. P., Caregiving in times of uncertainty: Helping adult children of aging parents find support during the COVID-19 outbreak (May 2020). | U.S.A. | Social Sciences | Commentary | Tablets |
| Luscombe, N., Morgan-Trimmer, S., Savage, S., & Allan, L., Digital technologies to support people living with dementia in the care home setting to engage in meaningful occupations: Protocol for a scoping review (June 2021). | U.K. | Health Sciences | Scoping review | Tablet devices, smartphones, robots, social media platforms |
| MacLeod, S., Tkatch, R., Kraemer, S., Fellows, A., McGinn, M., Schaeffer, J., & Yeh, C. S., COVID-19 era social isolation among older adults (May 2021). | U.S.A. | Health Sciences | Scoping review | Phone / web applications |
| Marsh, J. L., MSN, RN, O’Mallon, M., PhD, RN, Stockdale, S., PharmD, & Potter, D. R., PhD, APRN, PMHNP-BC, CAS., Caring for vulnerable populations during a pandemic: Literature review (Sept−Dec 2020). | U.S.A. | Health Sciences | Narrative review | Telehealth |
| Moore, K. J., Sampson, E. L., Kupeli, N., & Davies, N., Supporting families in end-of-life care and bereavement in the COVID-19 era. (April 2020). | U.K. | Health Sciences | Commentary | Electronic and online forms of communication |
| Office, E. E., Rodenstein, M. S., Merchant, T. S., Pendergrast, T. R., & Lindquist, L. A., Reducing social isolation of seniors during COVID-19 through medical student telephone contact (June 2020). | U.S.A. | Health Sciences | Qualitative research article | Phone |
| Pachana, N. A., Beattie, E., Byrne, G. J., & Brodaty, H., COVID-19 and psychogeriatrics: The view from Australia (October 2020). | Australia | Health Sciences | Commentary | Videoconferences |
| Pirhonen, J., Tiilikainen, E., Pekkarinen, S., Lemivaara, M., & Melkas, H., Can robots tackle late-life loneliness? Scanning of future opportunities and challenges in assisted living facilities (April 2020). | Finland | Social Sciences | Qualitative article | Robots |
| Rylett, R. J., Alary, F., Goldberg, J., Rogers, S., & Versteegh, P., Covid-19 and priorities for research in aging (August 2020). | Canada | Health Sciences | Commentary | Videoconferences, digital health services |
| Sacco, G., Lléonart, S., Simon, R., Noublanche, F., Annweiler, C., & TOVID Study Group, Communication technology preferences of hospitalized and institutionalized frail older adults during COVID-19 confinement: Cross-sectional survey study (Sept 2020). | France | Health Sciences | Quantitative research article | Phone, videocalls |
| Sano, M., Lapid, M. I., Ikeda, M., Mateos, R., Wang, H., & Reichman, W. E., Psychogeriatrics in a world with COVID-19 (June 2020). | U.S.A. | Health Sciences | Commentary | Computer, smartphone, Telehealth |
| Tsai, H.-H., Cheng, C.-Y., & Shieh, W.-Y., Effectiveness of laptop-based versus smartphone-based videoconferencing interaction on loneliness, depression and social support in nursing home residents: A secondary data analysis (Nov. 2020). | Taiwan | Health Sciences | Quantitative research article | Smartphone, laptop, telehealth |
| Veiga-Seijo, R., Miranda-Duro, M. D. C., & Veiga-Seijo, S., Strategies and actions to enable meaningful family connections in nursing homes during the COVID-19: A scoping review (June 2021). | Spain | Health Sciences | Scoping review | Information communication technologies (all) |
| Vernooij-Dassen, M., Verhey, F., & Lapid, M., The risks of social distancing for older adults: A call to balance (June 2020). | Netherlands | Health Sciences | Commentary | E-communications (all kinds) |
| Zamir, S., Hennessy, C., Taylor, A., & Jones, R., Intergroup ‘Skype’ quiz sessions in care homes to reduce loneliness and social isolation in older people (November 2020). | U.K. | Multidisciplinary | Qualitative research article | Skype through an iPad or television, e-health technologi |
